# Hypoxia and Cancer: From Bench to Bedside

**DOI:** 10.3390/cancers15092478

**Published:** 2023-04-26

**Authors:** Lingzhi Wang, Qingyu Zhou

**Affiliations:** 1 Cancer Science Institute of Singapore, National University of Singapore, Singapore 117599, Singapore; 2 Department of Pharmaceutical Sciences, Taneja College of Pharmacy, University of Florida, Tampa, FL 33612, USA

This Special Issue of *Cancers* (two original articles, five reviews), presented by international experts in tumor hypoxia, focuses on the role of hypoxia, or low oxygen levels, in the development and progression of cancer. This Special Issue includes seven papers that discuss various aspects of hypoxia in cancer, including its effects on tumor growth, metastasis, and resistance to therapy [1,2,3,4,5,6,7]. Davis et al. discussed the roles of the hypoxia-inducible factor (HIF) signaling pathway and the alpha subunits HIF-1α and HIF-2α in driving tumor progression in the hypoxic tumor microenvironment. While both subunits mediate the transcription of critical proteins that control oncogenic factors, HIF-2α remains under-considered in comparison to HIF-1α. This review highlights the spatiotemporal dynamics and roles of HIF-2α in promoting metastasis, tissue remodeling, angiogenesis, and upregulating cancer stem cell factors. It also discusses the potential for targeting HIF-2α as a novel strategy for solid cancer therapy [5]. Gkotinakou et al. discussed the interplay in cancer hypoxia focusing on vitamin D. They concluded that tumors have adaptive mechanisms that allow them to resist the anticancer effects of vitamin D, which are often attributed to the hypoxic microenvironment of solid tumors and the overexpression of hypoxia-inducible factors (HIFs). HIF-mediated signaling contributes to cancer cell survival, proliferation, and resistance to anticancer agents [4]. Janczy-Cempa et al. focused on cellular adaptation to hypoxic stress, characterized by an increase in oxidoreductase activity, which can be exploited in the design of hypoxia-activated prodrugs (HAPs) and fluorescent turn off–on probes. HAPs can be activated by oxidoreductases in hypoxic tumor cells, leading to targeted therapies that selectively kill tumor cells. Fluorescent turn off–on probes, on the other hand, can convert a non-fluorescent compound into a fluorescent one, specifically when imaging hypoxic cancer cells. This review highlights our current knowledge of the expression and activity of oxidoreductases that are relevant to the activation of HAPs and fluorescent imaging probes. It also discusses the current clinical status of HAPs, their limitations, and methods that may be used to improve their efficacy [3]. Hypoxia is also a critical feature of glioblastoma multiforme (GBM), a highly aggressive form of brain cancer. In a review article written by Park and Lee, several ways in which hypoxia contributes to GBM’s pathogenesis are summarized, including its promotion of resistance to conventional cancer therapies and inhibition of antitumor immune responses. As such, there is growing interest in the targeting of hypoxia as a therapeutic strategy for GBM. Given the recent success of immunotherapies in targeting a range of cancer types, it is increasingly apparent that an improved understanding of immune function within the GBM microenvironment and the role of hypoxia in mediating immune responses is necessary in order to improve GBM’s responsiveness to immunotherapies. This review provides an overview of hypoxia in GBM from clinical, pathological, and immunological perspectives, highlighting the need for further investigation of the interplay between hypoxia and the immune system in GBM [6]. McDonald et al. systematically summarized the progress of research on carbonic anhydrase IX (CAIX), a major protein involved in regulating pH and acidosis in tumor cells under hypoxic conditions, as a new potential target for cancer therapy [7]. This commentary discusses the current status of strategies targeting CAIX in both pre-clinical and clinical studies and highlights future perspectives on methods that leverage the inhibition of CAIX in combination with additional targeted therapies to create effective and durable approaches for cancer therapy.

Impressively, the study conducted by Godet et al. investigated the effect of post-hypoxic cells in promoting metastatic recurrence after chemotherapy treatment of triple-negative breast cancer (TNBC) and showed that TNBC cells that experience hypoxia in the primary tumor are resistant to chemotherapy at sites of metastasis [1]. The researchers utilized a hypoxia fate-mapping system to track the behavior of hypoxic cancer cells and found that post-hypoxic cells that metastasize to the lung and liver have decreased sensitivity to doxorubicin and paclitaxel but not cisplatin or 5-FU. This resistance to therapy leads to metastatic recurrence caused by post-hypoxic cells that are enriched in pathways related to cancer stem cell gene expression. This study highlights the importance of targeting hypoxia-induced cancer stem cells in TNBC to prevent treatment failure and relapse.

Interestingly, Chan et al. used the number of double-strand breaks (DSBs) to determine the effectiveness of proton beams in killing cancer cells [2]. They located cells at different depths along the path of the proton beams and used Monte Carlo simulations to estimate the DNA damage and repair outcomes. The study found that the proton beams were more effective in killing cancer cells than traditional photon therapy, especially under hypoxic conditions. The study also found that the effectiveness of the proton beams decreased as the energy level increased.

In summary, the role of hypoxia in cancer progression and the potential for targeting hypoxia as a strategy for cancer treatment, as well as the use of hypoxia-inducible factors as biomarkers for cancer diagnosis and prognosis, are intensively explored in this Special Issue. Overall, the papers highlight the importance of understanding the complex interplay between hypoxia and cancer in order to develop more effective therapies for this challenging disease.
